# Prospective observation and merging of motor vehicle accident data with patient treatment data - First-time data merging for the TR-DGU^®^

**DOI:** 10.1007/s00068-025-02872-0

**Published:** 2025-05-12

**Authors:** Bastian Brune, Maximilian Wolf, Daniel Stappert, Sascha Keil, André Nohl, Frank Herbstreit, Oliver Kamp, Dan Bieler, Lars Becker, Thorsten Brenner, Christian Waydhas, Marcel Dudda

**Affiliations:** 1 Department of Trauma, Hand and Reconstructive Surgery, University Hospital Essen, University Duisburg-Essen, Essen, Germany; 2 Directorate of Emergency Medical Services, Fire Department Essen, Essen, Germany; 3 Fire Department Essen, Essen, Germany; 4 Emergency Department, BG-Klinikum Duisburg, Duisburg, Germany; 5 Directorate of Emergency Medical Services, Fire Department Oberhausen, Oberhausen, Germany; 6 Department of Anesthesiology and Intensive Care Medicine, University Hospital Essen, University Duisburg-Essen, Essen, Germany; 7 Department of Trauma Surgery and Orthopaedics, Reconstructive and Hand Surgery, Burn Medicine, Germany Armed Forces Central Hospital Koblenz, Koblenz, Germany; 8 Department of Orthopaedics and Trauma Surgery, BG Klinikum Duisburg, Duisburg, Germany

## Introduction

This article describes the merging of datasets from several data sources from an accident event to preclinical treatment, further clinical care, and the resulting evaluation in German Society for Trauma Surgery’s (DGU) TraumaRegister (TR-DGU^®^) in a prospective observational study. The datasets used for merging are stored in databases of an emergency medical service (EMS) and a hospital. The potential of data merging is explained on the first case in which it was possible to trace the accident event to the TR-DGU^®^.

### Current data flow into the TR-DGU^®^

For over 30 years, the DGU has been evaluating the course of treatment from accident reports to hospital treatment in the TR-DGU^®^. The datasets are entered manually by the personnel of participating hospitals. The registry information and associated research results influence national treatment concepts and can be described as groundbreaking for traumatological emergency medicine [1].

Since the update of the German Guideline on the Treatment of Patients with Multiple and/or Severe Injuries in Germany in late 2022, fewer motor vehicle accident (MVA) mechanisms are an indication for trauma team activation. The trauma team activation is now based even more on vital parameters and signs of injury (primary criteria) than accident mechanism-related reasons (secondary criteria). However, the accident data documented in the TR-DGU^®^, which were also the basis for the re-evaluation of the indicators of trauma team activation, are based on subjective information provided by the EMS personnel or people involved in the accidents. Conclusions about the severity of injury are therefore also based on subjective information. Accurate and objective information about the mechanism of accidents and the circumstances surrounding it could lead to the development of more reliable criteria for trauma team activation.

### Accident data

The European Regulation 2015/758 stipulates that new vehicles must be equipped with an automatic emergency call system. EmergencyCall systems (eCall systems) have been mandatory in Germany for new models of passenger cars and light commercial vehicles since 04/01/2018. Standards have been established for general requirements for in-vehicle emergency call systems, components and autonomous technical units. The prerequisites for the operational capability of an eCall system in a passenger car are shown in Table 1.

**Table 1:** Requirements for operating an eCall system

GPS receiver for determining the position of the vehicle
GSM antenna for sending the emergency call
Control unit for reporting the location
Crash sensor for detecting the type of accident
Hands-free system
Separate emergency power supply
Button for manually triggering the emergency call
Indicator light

In Germany, accident data can be transmitted via two systems to the public safety answering points (PSAP). These are eCall systems via the emergency call number 112 and so-called third-party service eCalls (TPS-eCalls). ECalls can be triggered manually or automatically to provide vehicle occupants with early assistance in medical emergencies. Both systems contain a minimum set of data (MSD) (Table 2). TPS-eCall systems optionally contain further data (Table 3). Due to increasing demands in the European New Car Assessment Program (Euro NCAP), even further data availability is to be expected. Depending on the manufacturer, data such as airbag deployment, number of passengers and information on changes in velocity are recorded and might be available in the future. Information can be forwarded by telephone or based on datasets. The development of evidence-based protocols for the effective use of telemetry data from motor vehicles has been discussed for a long time.

**Table 2:** Parameters of the minimal set of data (= MSD)

Element des Minimaldatensatzes (MSD)	Angabe
ECall activation	Automatic or manual activation
Test-Call	Test use or real use
Plausible position information	Yes/ no
Vehicle type	Type of vehicle
VIN	Vehicle identification number
Drive type	Petrol, diesel, hybrid, electric drive
Time information	Time of activation
Position Latitude	Location at the time of activation (Latitude)
Position Longitude	Location at the time of activation (Longitude)
Direction of travel	Last direction of travel

**Table 3:** Comparison of eCall-Systems

	112-eCall-System	TPS-eCall-System
First contact	PSAP*	Service Center**
Data transmission	yes	possible
Data set	MSD	MSD + optional data
Costs for vehicle owner	None	possible
Time of accident	yes	yes
Trigger type	yes	yes
Vehicle identification number	yes	yes
Drive type	yes	yes
Vehicle position (geodata)	yes	yes
Direction of travel	yes	yes
Number of occupants	yes	yes

* Contact is only made in the event of relevant accidents (airbag deployment)

** The service center is contacted after every accident. The service center forwards the callouts if indicated

## Potential of data merging

ECall systems and their implementation in the rescue chain represent a way of forwarding objective accident data automatically from the vehicle manufacturer to the rescue service personnel and to the TR-DGU^®^ within seconds. It should be noted here that with increasing safety standards, further parameters, such as the use of emergency braking assistance systems or velocity changes during the car accident could be transmitted.

## Aim of the study

The aim of our study was to evaluate MVAs in the city of Essen in the first half of the year 2023 (01/01/2023 to 06/30/2023) and to examine in which cases a TPS-eCall data record was available that could potentially provide further information on the accident event. We also investigated whether a link to the TR-DGU^®^ could be achieved.

## Methods

This is a prospective observational study of treatment protocols from a PSAP, an EMS and a hospital. One author (BB) works at the participating hospital and is also a member of the medical management of the emergency medical service (EMS). In the context of professional supervision, an overall assessment of the treatment data from the EMS and the hospital can be made.

The data required for the analysis are available in different databases. The data were collected independently by the PSAP and the hospital and merged in a multi-stage process. The merging process links the call logs of the PSAP including attached eCall-protocols, the EMS protocols and the medical documentation of the hospital. All EMS operations from 01/01/2023 to 06/30/2023 for which the alert was raised by a TPS-eCall service center were considered. Emergency calls that were received by the PSAP via 112-eCalls could not be identified in the merging process.

Keywords for the terms “car”, “vehicle” and “traffic accident” were gradually filtered out of all the EMS protocols. The protocols were then linked to the call logs. In the call logs of the PSAP, alarms from the TPS-eCall service center were filtered out and linked to the database of the EMS. The data were categorized into TPS-eCall and non-TPS-eCall operations. Operations in which the reasons for the call indicated non-trauma-related reasons for the alarm were not included in the evaluation. For operations with multiple protocols, e.g. due to multiple patient treatments, information from all protocols was evaluated. In a further step, if a patient was transported to the participating hospital and consented to take part in the study, the medical documentation and TPS-eCall data records was also checked. Patients who were transported to other hospitals could not be further evaluated.

## Results

**Fig. 1:**
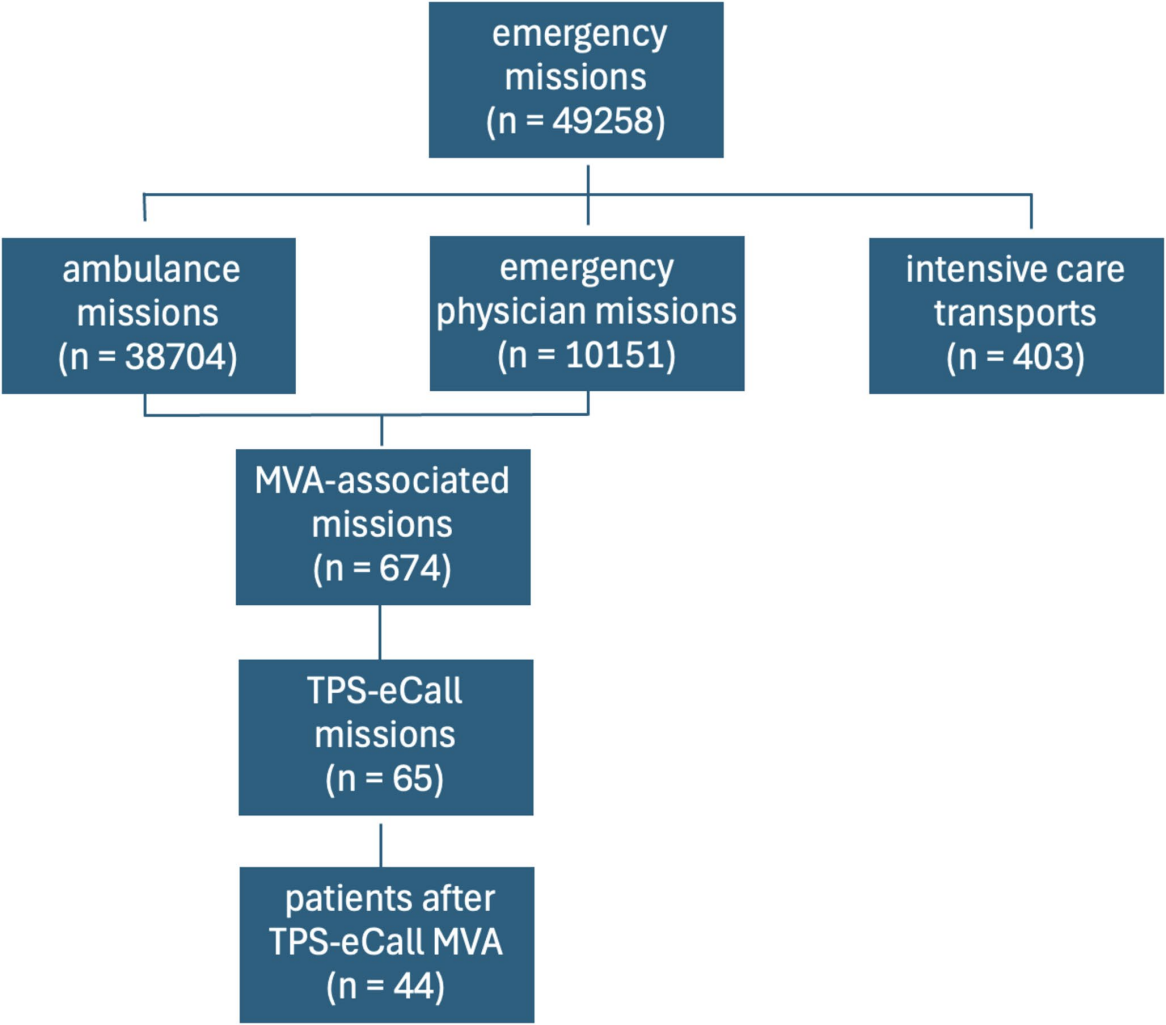
Emergency missions

A total of 49,258 emergency operations (38,704 emergency medical operations, 10,151 emergency physician (EP) operations, 403 intensive care transports) were carried out from 01/01/2023 to 06/30/2023. 239 EP callouts and 776 ambulance callouts were initiated after 647 MVA. Of all emergency calls in the city of Essen during the study period, 65 MVA (10%) were associated with TPS-eCalls. In 44 cases, rescue operations were carried out (Fig. 1). In 21 cases the PSAP was contacted to ensure traffic safety because of damaged vehicles. The EMS relevant TPS-eCall-accidents to non-TPS-eCall-accidents was 6.8%.

**Fig. 2:**
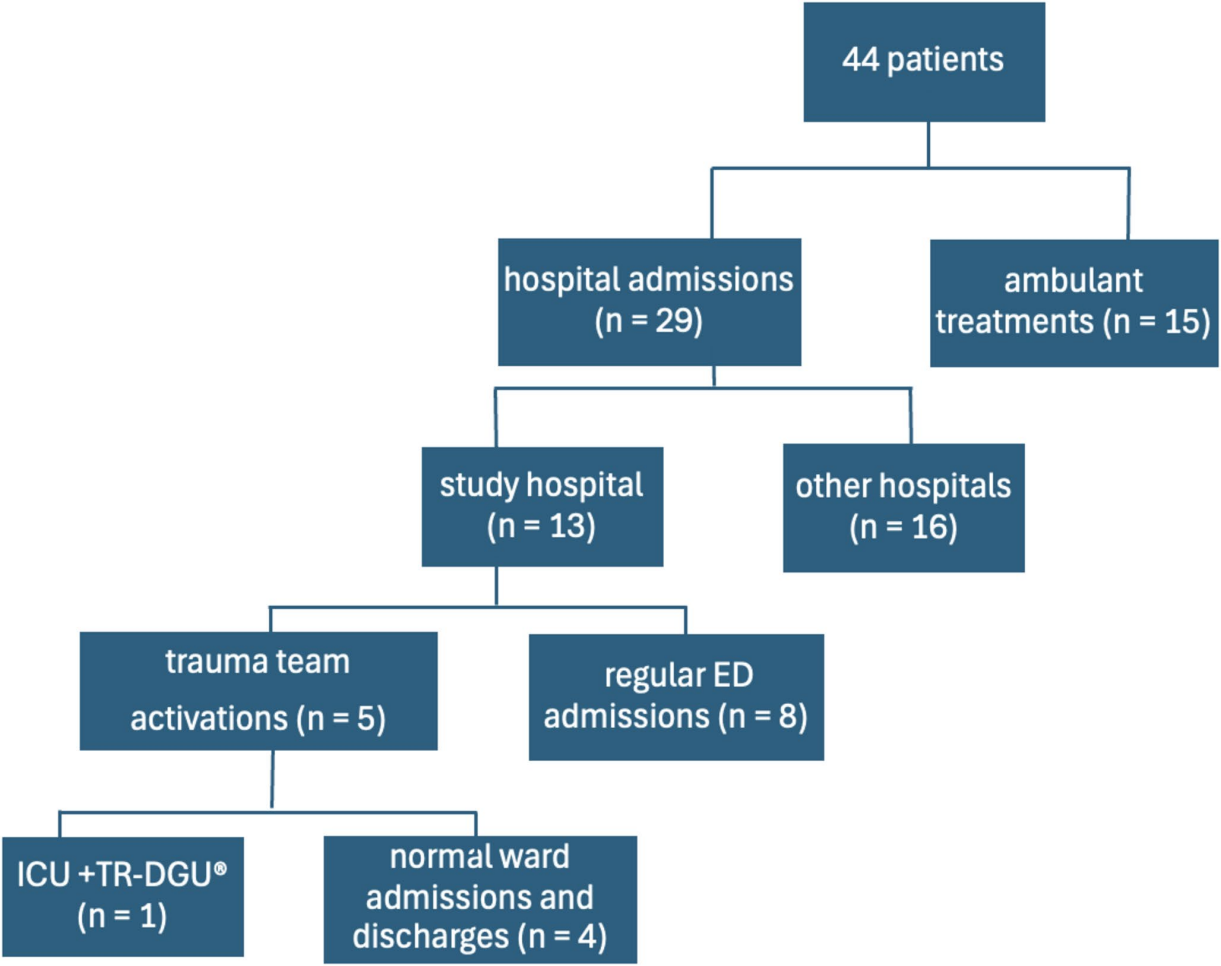
Patient flow chart

In the remaining 44 rescue operations, 29 people were transported to hospitals (Fig. 2). 13 of them were transported to the study hospital and 16 to other hospitals. Due to the preclinical assessed injury severity of the patients transported to the study hospital, 5 had to be treated by the trauma team. 8 were treated in the regular emergency room. One of the patients treated by the trauma team subsequently required intensive care. This was the first case in which the datasets from the accident data to the consequences of the accident, including the data from the treatment protocols of the ambulance service and the hospital, could be successfully linked to the TR-DGU^®^.

**Fig. 3:**
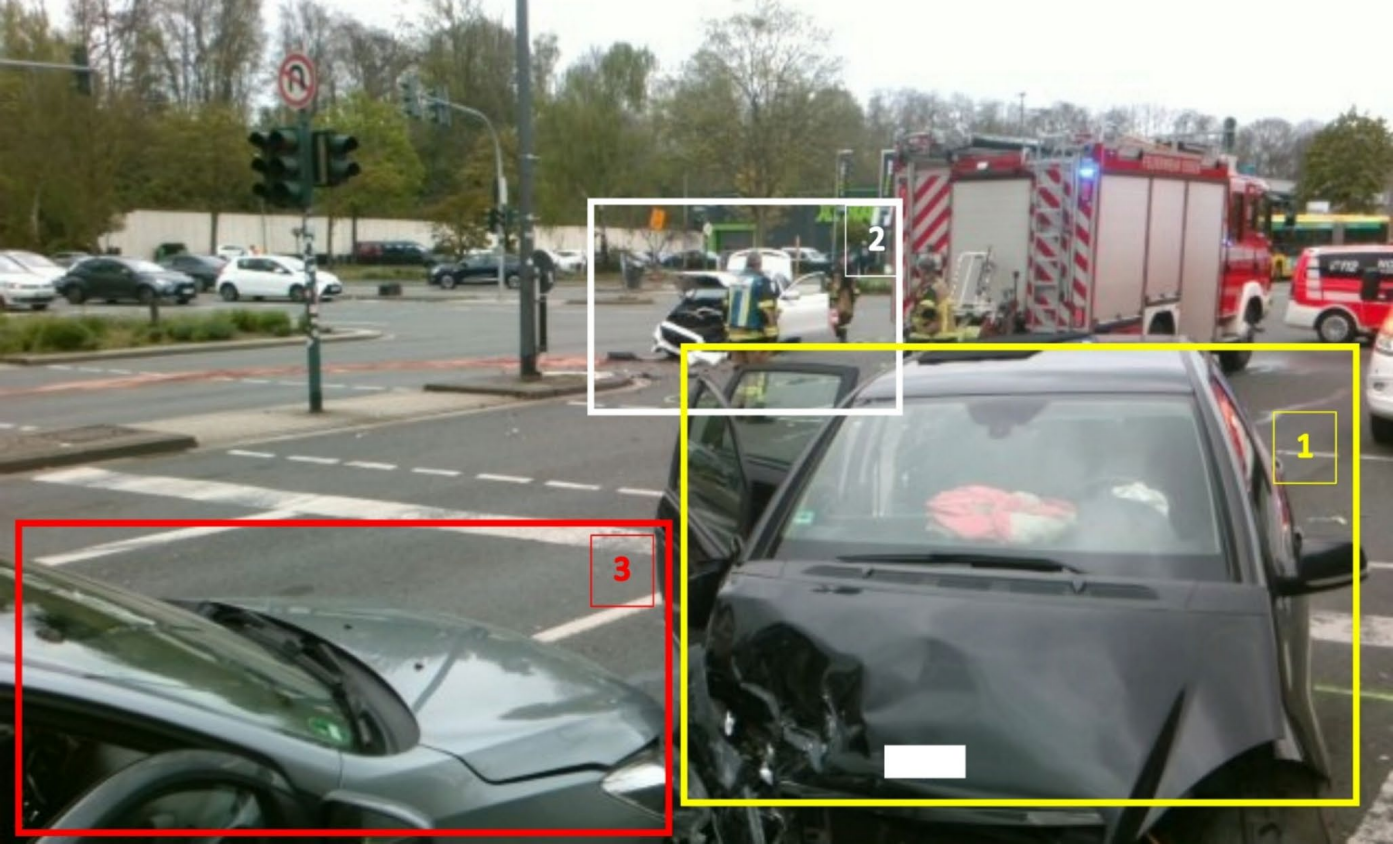
Accident scene. Frame colors: yellow: vehicle 1 (seriously injured passenger), white: vehicle 2 (TPS-eCall alarm), red: vehicle 3

A detailed examination of the accident revealed that three vehicles, each with one occupant, were involved. To describe the potential of eCall data application for understanding accident events, the data from the EMS treatment protocols of two patients are presented first. A third person involved in the accident did not request treatment by the EMS. The anonymized eCall data set and a photograph of the scene of the accident can be found in Fig. 3 (Fig. 3) and Table 4 (Table 4).

**Table 4:** ECall dataset

Position	51.4XXXX, 6.9XXXX **
Location N-1	51,4XXXX, 6,9XXXX **
Location N-2	51,4XXXX, 6,9XXXX **
Drive type	Diesel
Control	Automatic
Position Confidence	CAN_BE-Trusted
Vehicle type	Passenger_car_M1
Vehicle identification number	XXXXXXXXXXXXXXXX **
Event time (UTC):	TT.MM.JJJJ hh: mm: ss **
Vehicle direction:	0
Number of passengers	0*

*: last entry after the occupant exited the vehicle

**: Data relating to persons, persons and accidents have been anonymized

### Vehicle 1 (2 collisions, no eCall installation)

1 patient (68-year, female): emergency call from the TPS eCall-service center: 1:05 p.m. Alerting of ambulance & EP: 1:08 p.m. Arrival of first ambulance at 1:14 p.m. Head-on collision at 30–60 km/h. Seat belt used. Airbags deployed. First frontal impact on the right side, then the car skidded and collided head-on with a third car. Technical rescue.

Hospitalization of the patient for 11 days, including 4 days in intensive care. Serial rib fractures with stable thorax and lung contusion, intial reduction in blood oxygen concentration, distal forearm shaft fracture, multiple lacerations and abrasions. ISS: 13, NISS: 17, GCS: 15, RTS: 7.841, TASH: 0, RISC II: 99.5%, TRISS: 96.6%. Entry in the TR-DGU^®^. TraumaRegister No. 2023-XXX (personal and accident-related data have been anonymized).

Vehicle 2 (one collision, triggered the eCall): 1 patient (28-year, male): eCall: 1:04 p.m., eCall by TPS-service center: 1:05 p.m. Alerting ambulance & EP: 1:08 p.m. Arrival of the first ambulance: 1:15 p.m. Frontal impact on the side of another car. Airbag and seat belt deployed. Velocity 30–60 km/h. Self-mobilization. Outpatient admission to external hospital for minor retrograde amnesia and shoulder contusion.

Vehicle 3 (second collision, no eCall installation): 1 occupant (unknown). No EMS intervention. No EMS protocol.

## Discussion

ECall systems promise to transmit the exact time, place and mechanism of accidents, and thus to improve both the dispatching of the PSAPs and the assessment of the EMS personnel. As a result, the preclinical treatment can be shortened. The authors are not aware of a structured analysis of the vehicles equipped with eCall technology among those involved in accidents. In the current study, the proportion of vehicles equipped with TPS-eCall technology is 10%. Due to the legal requirement only established in 2018, a further increase in this percentage is expected in the coming years. In addition, there are112-eCall data, which cannot be included in the current study. The proportion of eCall data that could potentially be used for medical purposes is therefore considered relevant.

The potential improvement is most obvious when vehicle occupants are unable to make an emergency call due to (accident-related) loss of consciousness and their accident is not noticed by other people. Due to a lower population density, such situations occur more frequently in rural areas, with delays of more than five minutes in 20% of cases [2, 3]. However, unnoticed accidents are less common in Central Europe and tend to be characterized by more complex accident mechanisms at intersections, involving several vehicles. In these cases, the complexity of accident assessment increases for all persons involved in patient care from PSAP to hospital personnel. Due to problems in assessing the severity of an accident and its consequences, do over- and undertriage not only occur in the clinical treatment, but also previously at the PSAP and the EMS.

Undertriage of the PSAP leads to delays until the first EMS treatment. In view of the advantages of automatic alerting and the resulting possibility of the EMS arriving at short notice, the advantages of a fast and objective accident report via TPS-eCall become clear. For example, post-hoc analyses showed a reduced need for intervention if the EMS arrive early for initial treatment [4]. Estimates of the potential reduction in the number of MVA-associated deaths due to the early treatment of reversible causes of death made possible by the use of automatic eCall systems range from 2 to 15% in Europe [2].

In MVA accidents, however, an overtriage of the EMS follows the undertriage of the PSAP [5]. Due to overtriaging, a large proportion of kinematic indicators for trauma team activation have not been considered since the guideline update for the care of polytrauma patients of the DGU in 2022. The only accident pattern that remains is ejection from the vehicle [1]. Indicators such as a change of velocity of more than 30 km/h and a deformation of the car body of more than 50 cm were removed from the list of trauma team activation indicators.

In the case of our patient neither the frontal-side accident mechanism nor the consequences of the accident (serial rib fractures without thoracic instability, lung contusion and forearm shaft fracture) were sufficient to justify treatment of the trauma team according to the new guidelines.

**Fig. 4:**
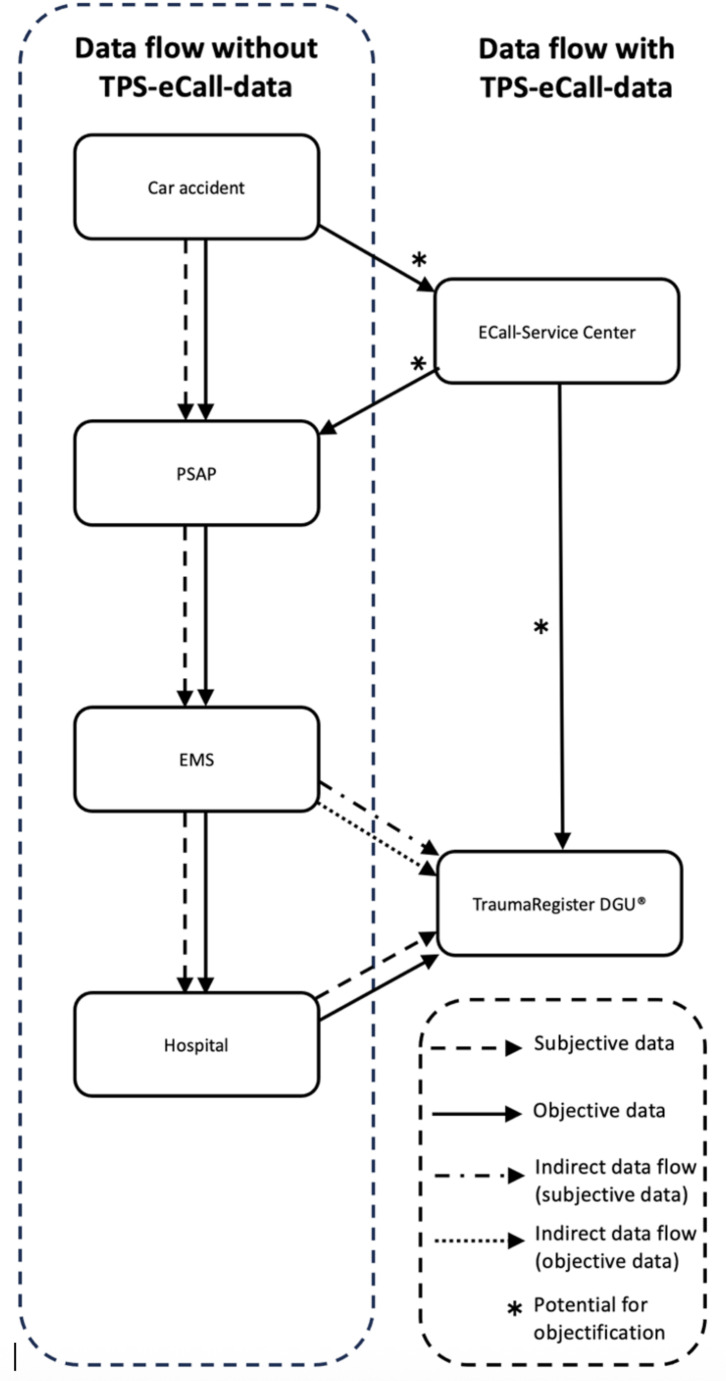
Rescue chain plus potential for objectification and additional data flow

In the accident described, the trauma team was alerted because of a brief reduction in oxygen saturation in the blood as a sign of the lung contusion and also because of an estimated velocity of 50 km/h, in accordance with the former guideline. According to the new guideline, in addition to the direction of the accident already mentioned, velocity alone would not have been sufficient to justify the activation of the trauma team. Both, the categorization side impact and the differences in velocity of vehicle 2 could support the decision to activate the trauma team for patient 1. This is particularly evident in the accident described with different accident mechanisms for 3 cars. A possible solution for integrating objective accident data into the data flow of the rescue chain is shown in Fig. 4. If the data flow proposed in Fig. 4 is established and an increasing number of accident data and treatment data can be merged, the evaluation of these data could provide insights into accident mechanisms and their consequences. However, an appropriate definition of accident mechanisms, which in our case are different for all parties, is essential for the assessment, if accident data are used [6].

In our case, the PSAP alerted 2 ambulances and 1 EP. The dispatchers’ assessment therefore led to the correct number of EMS resources according to the documentation. If this was due to the statements of the TPS-service center can not be determined. As at least 3 people were involved in the accident a need for discussion remains. Despite the EMS` documentation of a head-on and head-on accident, it was a primary head-on and side accident. The assessment of the accident event was therefore definitely flawed. The information about the side impact, ideally with a description of the position of patient 2 on the driver’s side, which also corresponded to the side of impact, would have improved the assessment of the attending personnel of the PSAP and the EMS. Fortunately, the misjudgment in the described case had no negative impact on the treatment outcome. Due to the resulting injuries trauma team activation and intensive care treatment followed for patient 1. The head-on impact of vehicle 2/ patient 2 resulted in regular treatment in the emergency room. The third party with a head-on impact, with presumably lower velocity due to a secondary collision, stated that no treatment was necessary.

In the case described the details of the time of the accident and the direction of travel of one of the parties involved were specified. In the future, a qualitative increase of accident information is to be expected with the expansion of the transmitted data. The mandatory installation of eCall systems also ensures a quantitative expansion of the system. Situations like the current example, in which patient 1, whose case was entered in the TR-DGU^®^ was in a vehicle that did not transmit any accident data, will become less common in the long term. Developments are more likely to lead to accident events with data transmission from all vehicles involved. In this case, the transmission of extended data from vehicle 2 would have led to more accurate assessment. Unfortunately, due to the lack of data on the change in driving velocity, the type of impact and the number of occupants in the MSD, only the exact place and time direction of travel of vehicle 2 was transmitted. The direction coincides with the point of impact of vehicle 1 and the injury pattern of the patients with serial rib fractures, lung contusions and a forearm fracture, but in total only limited additional information was obtained. Nevertheless, preparations should be made to incorporate the additional information into the rescue chain and also the TR-DGU^®^. A detailed examination of the crash data, particularly the forces acting and their locations on the body and the patient, can provide new insights when considering the patient’s position in the vehicle. However, objectification of accident locations and real-time alerts from PSAP can also have a positive impact on the outcome. In addition to the existing subjective data, Fig. 4 also shows a flow of objective TPS-Call-data and their availability at the following points from MVA to the TR-DGU^®^.

## Conclusion

Merging objective accident data with the medical documentation of a seriously injured patient from a MVA to the TR-DGU^®^ was possible. In the present case, the accident data was indirectly transmitted by vehicle 2, while the patient’s vehicle 1 did not transmit any data. Due to the obligation to install eCall systems in passenger cars, an increasing number of eCall-alerts are to be expected. We assume that the recordings of all parties involved in the accident will be available together and thus a statement about the exact time of accident and all directions of movement, airbag deployment, as well as differences in velocity will be available.

The combination of eCall data and medical data will pose major challenges for emergency medicine but will also offer enormous potential for development. The use of objective accident data offers the potential for future improvements, from alerting the EMS earlier, possibly with a known number of people involved in the accident and thus knowledge of the necessary rescue resources. In the future, the standardized transmission of eCalls to PSAPs and EMS will therefore offer the opportunity to objectify accident mechanisms in hospital treatment and in the TR-DGU^®^ and to subsequently make register-based treatment recommendations.

### Limitations

The current study is subject to numerous limitations. The prospective observational study focuses on comparatively low case numbers from a short time interval. This applies in particular because only one seriously injured person was recorded. Comparative calculations on the outcome of accident victims after different TPS-eCall-associated MVA have not been possible. The prospective data on accident velocity was not provided by the automobile manufacturer in the described case. The planned change to the ESD standardization, as well as an optimization of the data connection between the vehicle triggering the eCall, the PSAP, the EMS, the hospital and the TR-DGU^®^, which is currently not available across the board, can have a positive effect on data availability and data flow.

## Data Availability

The data analyzed in the study can be obtained from the author in anonymized form upon reasoned request and with the permission of the data owner. The personal rights of the persons involved in the accident must be preserved.

## Abbreviations

DGU: German Trauma Society
ECall: EmergencyCall
EMS: Emergency Medical Service
EP: Emergency Physician
Euro NCAP: European New Car Assessment Program
GCS: Glasgow Coma Scale
GPS: Global Positioning System
GSM: Global System for Mobile Communications
ISS: Injurity Severity Score
MVA: Motor vehicle accident
NISS: New Injurity Severity Score
PKW: Passenger Car
PSAP: Public Safety Answering Point
RISC II: Revised Injurity Severity Score (Version II)
RTS: Revised Trauma Score
TASH: Trauma Associated Severe Hemorrhage
TPS: Third Party Service
TR-DGU®: TraumaRegister of the German Trauma Society^®^
TRISS: Trauma Score and Injurity Severity Score
UTC: Coordinated Universal Time
